# Supplementary material for: How populist attitudes scales fail to capture support for populists in power
Source: PLoS One. 2021 Dec 31;16(12):e0261658. doi: 10.1371/journal.pone.0261658 (PMC8719679; doi:10.1371/journal.pone.0261658)
Supplement: S1 File — (PDF) [file pone.0261658.s001.pdf]

Supplementary Information for
"How populist attitude scales fail to capture support for populists in power" published in *PLOS ONE*

Sebastian Jungkunz[1,2,3,*], Robert A. Fahey[4,*], Airo Hino[5],

**1** Institute for Socio-Economics, University of Duisburg-Essen, Duisburg, Germany
**2** Chair of Political Sociology, University of Bamberg, Bamberg, Germany
**3** Center for Political Communication, Zeppelin University, Friedrichshafen, Germany
**4** Waseda Institute of Political Economy, Waseda University, Tokyo, Japan
**5** School of Political Science and Economics, Waseda University, Tokyo, Japan

# Contents

**Appendix A: Main Study** **2**

**Appendix B: Robustness Checks** **13**
Greece (2016) . . . . . . . . . . 13
Japan (2019) . . . . . . . . . . 16

# Appendix A: Main Study

**Table S1.** Overview CSES Data and Populist Parties

| Country | Year | N | Populist Parties | In Power before election |
|---|---|---|---|---|
| Austria | 2017 | 1203 | FPÖ | no |
| Australia | 2017 | 2000 | – | – |
| Belgium (Flanders) | 2019 | 1084 | Vlaams Belang | no |
| Belgium (Wallonia) | 2019 | 730 | – | – |
| Brazil | 2018 | 2506 | PSL | no |
| Canada | 2019 | 2889 | – | – |
| Chile | 2017 | 2000 | – | – |
| Costa Rica | 2019 | 1456 | – | – |
| Finland | 2019 | 1598 | Finns Party | no |
| France | 2017 | 1830 | FN, LFI | no, no |
| Germany | 2017 | 2032 | AfD, Left Party | no, no |
| Great Britain | 2017 | 2194 | – | – |
| Greece[1] | 2015 | 1078 | SYRIZA | no |
| Hong Kong[1] | 2016 | 1020 | – | – |
| Hungary | 2018 | 1208 | FIDESZ, Jobbik | yes, no |
| Iceland | 2016 | 1295 | People's Party | no |
| Iceland | 2017 | 2073 | Centre Party, People's Party | no, no |
| Ireland[1] | 2016 | 1000 | Sinn Féin | no |
| Italy | 2018 | 2001 | FI, LN, M5S | no, no, no |
| Japan | 2017 | 1688 | (LDP)[2] | yes |
| Lithuania | 2016 | 1500 | Order and Justice | no |
| Montenegro | 2016 | 1213 | DPS | yes |
| Netherlands | 2017 | 3428 | FvD, PVV, SP | no, no, no |
| New Zealand | 2017 | 1808 | – | no |
| Norway | 2017 | 1792 | FrP | no |
| Portugal | 2019 | 1500 | – | – |
| South Korea[1] | 2016 | 1199 | (Saenuri)[2] | yes |
| Sweden | 2018 | 3784 | Sweden Democrats | no |
| Switzerland | 2019 | 4645 | SVP | no |
| Taiwan[1] | 2016 | 1690 | – | – |
| Taiwan | 2020 | 1680 | – | – |
| Turkey | 2018 | 1069 | AKP | yes |
| United States | 2016 | 3648 | – | – |
| United States | 2020 | 8280 | – | – |

Populist parties that gained less than five percent of the popular vote are not listed as they were omitted from the analysis due to computational reasons (low sample size).
"In Power" indicates whether a populist party has been the ruling party already prior to the respective election survey.
[1] Part of the CSES pre-test with reversed E3004_1 item.
[2] Neither the LDP nor Saenuri are formally classified as populist parties, and we do not treat them as such in this study. However, each of them has been argued to have adopted elements of populist rhetorical strategy.

**Table S2.** Confirmatory Factor Analysis Models (3 Dimensions)

| **Country** | **N** | **RMSEA** | **SRMR** | **CFI** | **Avg. Loading** | **Min. Loading** | **Lowest Loading** |
|---|---|---|---|---|---|---|---|
| Austria | 1020 | 0.068 | 0.024 | 0.982 | 0.679 | 0.701 | E3004_4 |
| Australia | 1666 | 0.054 | 0.022 | 0.985 | 0.751 | 0.550 | E3004_3 |
| Belgium (Flanders) | 922 | 0.091 | 0.028 | 0.967 | 0.7605 | 0.534 | E3004_3 |
| Belgium (Wallonia) | 583 | 0.103 | 0.034 | 0.950 | 0.754 | 0.549 | E3004_3 |
| Brazil | 1933 | 0.031 | 0.018 | 0.984 | 0.620 | 0.201 | E3004_3 |
| Canada | 1974 | 0.050 | 0.019 | 0.982 | 0.710 | 0.497 | E3004_3 |
| Chile | 1324 | 0.078 | 0.038 | 0.914 | 0.492 | 0.250 | E3004_3 |
| Costa Rica | 1030 | 0.041 | 0.022 | 0.976 | 0.650 | 0.312 | E3004_3 |
| Finland | 902 | 0.070 | 0.025 | 0.979 | 0.642 | 0.484 | E3004_3 |
| France | 1286 | 0.072 | 0.029 | 0.968 | 0.627 | 0.518 | E3004_3 |
| Germany | 1659 | 0.024 | 0.010 | 0.998 | 0.653 | 0.541 | E3004_3 |
| Great Britain | 781 | 0.106 | 0.036 | 0.946 | 0.731 | 0.448 | E3004_3 |
| Greece[1] | 717 | 0.082 | 0.035 | 0.929 | 0.587 | 0.420 | E3004_3 |
| Hong Kong[1] | 722 | 0.066 | 0.038 | 0.811 | 0.470 | 0.190 | E3004_3 |
| Hungary | 832 | 0.079 | 0.031 | 0.963 | 0.634 | 0.435 | E3004_3 |
| Iceland 2016 | 848 | 0.055 | 0.023 | 0.983 | 0.770 | 0.679 | E3004_3 |
| Iceland 2017 | 1381 | 0.044 | 0.018 | 0.989 | 0.766 | 0.604 | E3004_3 |
| Ireland[1] | 827 | 0.036 | 0.016 | 0.992 | 0.602 | 0.482 | E3004_3 |
| Italy | 1260 | 0.082 | 0.034 | 0.941 | 0.583 | 0.398 | E3004_3 |
| Japan | 1352 | 0.061 | 0.027 | 0.958 | 0.584 | 0.462 | E3004_3 |
| Lithuania | 1008 | 0.101 | 0.045 | 0.916 | 0.612 | 0.408 | E3004_3 |
| Montenegro | 806 | 0.062 | 0.026 | 0.978 | 0.589 | 0.158 | E3004_3 |
| Netherlands | 2355 | 0.074 | 0.022 | 0.979 | 0.780 | 0.585 | E3004_3 |
| New Zealand | 1290 | 0.064 | 0.025 | 0.977 | 0.745 | 0.488 | E3004_3 |
| Norway | 1583 | 0.052 | 0.021 | 0.984 | 0.748 | 0.589 | E3004_3 |
| Portugal | 1152 | 0.058 | 0.028 | 0.968 | 0.710 | 0.445 | E3004_3 |
| South Korea[1] | 1179 | 0.042 | 0.024 | 0.960 | 0.539 | 0.425 | E3004_3 |
| Sweden | 3170 | 0.086 | 0.026 | 0.974 | 0.682 | 0.422 | E3004_3 |
| Switzerland | 3826 | 0.036 | 0.015 | 0.992 | 0.721 | 0.383 | E3004_3 |
| Taiwan 2016[1] | 1248 | 0.062 | 0.031 | 0.935 | 0.555 | 0.411 | E3004_4 |
| Taiwan 2020 | 1350 | 0.046 | 0.024 | 0.969 | 0.655 | 0.408 | E3004_3 |
| Turkey | 912 | 0.068 | 0.030 | 0.954 | 0.692 | 0.426 | E3004_3 |
| United States 2016 | 3481 | 0.061 | 0.024 | 0.970 | 0.607 | 0.457 | E3004_3 |
| United States 2020 | 6734 | 0.070 | 0.026 | 0.967 | 0.695 | 0.480 | E3004_6 |

[1] Part of the pre-test with reversed E3004_1 item. As the Swedish data does not contain E3004_1, we used a two-factor model without a Manicheanism dimension. Shown are standardized loadings.

**Table S3. Confirmatory Factor Analysis Models Wuttke et al. version (two dimensions)**

| Country | N | RMSEA | SRMR | CFI | Avg. Loading | Min. Loading | Lowest Loading |
|---|---|---|---|---|---|---|---|
| Austria | 986 | 0.082 | 0.030 | 0.961 | 0.650 | 0.452 | E3004_6 |
| Australia | 1638 | 0.076 | 0.040 | 0.949 | 0.568 | 0.125 | E3005_2 |
| Belgium (Flanders) | 916 | 0.066 | 0.029 | 0.970 | 0.634 | 0.477 | E3005_2 |
| Belgium (Wallonia) | 574 | 0.060 | 0.032 | 0.968 | 0.570 | 0.243 | E3005_2 |
| Brazil | 1898 | 0.051 | 0.021 | 0.977 | 0.432 | 0.199 | E3005_2 |
| Canada | 1822 | 0.052 | 0.025 | 0.970 | 0.544 | 0.336 | E3005_2 |
| Chile | 1311 | 0.046 | 0.032 | 0.904 | 0.418 | 0.050 | E3005_2 |
| Costa Rica | 1018 | 0.027 | 0.022 | 0.965 | 0.293 | 0.020 | E3005_2 |
| Finland | 864 | 0.053 | 0.037 | 0.977 | 0.592 | 0.354 | E3005_2 |
| France | 1406 | 0.089 | 0.045 | 0.908 | 0.503 | 0.118 | E3005_2 |
| Germany | 1592 | 0.062 | 0.030 | 0.975 | 0.627 | 0.378 | E3005_2 |
| Great Britain | 702 | 0.100 | 0.047 | 0.921 | 0.689 | 0.312 | E3005_2 |
| Hungary | 813 | 0.068 | 0.043 | 0.915 | 0.490 | 0.075 | E3005_2 |
| Iceland 2016 | 805 | 0.067 | 0.034 | 0.958 | 0.519 | 0.090 | E3005_2 |
| Iceland 2017 | 1275 | 0.069 | 0.032 | 0.956 | 0.528 | 0.086 | E3005_2 |
| Italy | 1268 | 0.074 | 0.039 | 0.911 | 0.507 | 0.332 | E3004_1 |
| Japan | 1234 | 0.051 | 0.030 | 0.928 | 0.357 | −0.013 | E3005_2 |
| Lithuania | 994 | 0.090 | 0.047 | 0.873 | 0.474 | 0.131 | E3004_1 |
| Montenegro | 786 | 0.086 | 0.045 | 0.918 | 0.490 | 0.182 | E3004_3 |
| Netherlands | 1518 | 0.055 | 0.025 | 0.979 | 0.629 | 0.379 | E3005_2 |
| New Zealand | 1099 | 0.064 | 0.032 | 0.963 | 0.565 | 0.270 | E3005_2 |
| Norway | 1577 | 0.055 | 0.026 | 0.972 | 0.563 | 0.325 | E3005_2 |
| Portugal | 1096 | 0.069 | 0.040 | 0.904 | 0.447 | 0.070 | E3007 |
| Switzerland | 3799 | 0.068 | 0.035 | 0.956 | 0.673 | 0.400 | E3004_3 |
| Turkey | 729 | 0.073 | 0.043 | 0.899 | 0.335 | −0.075 | E3005_2 |
| United States 2016 | 3448 | 0.073 | 0.040 | 0.930 | 0.538 | 0.370 | E3005_2 |
| United States 2020 | 6693 | 0.074 | 0.038 | 0.936 | 0.571 | 0.301 | E3005_2 |

Shown are standardized loadings.

**Fig S1. Predictions of Populist Vote Across Countries (3 Dimensions) I**
Predictions of vote choice are based on multinomial structural equation models with 95% confidence intervals.

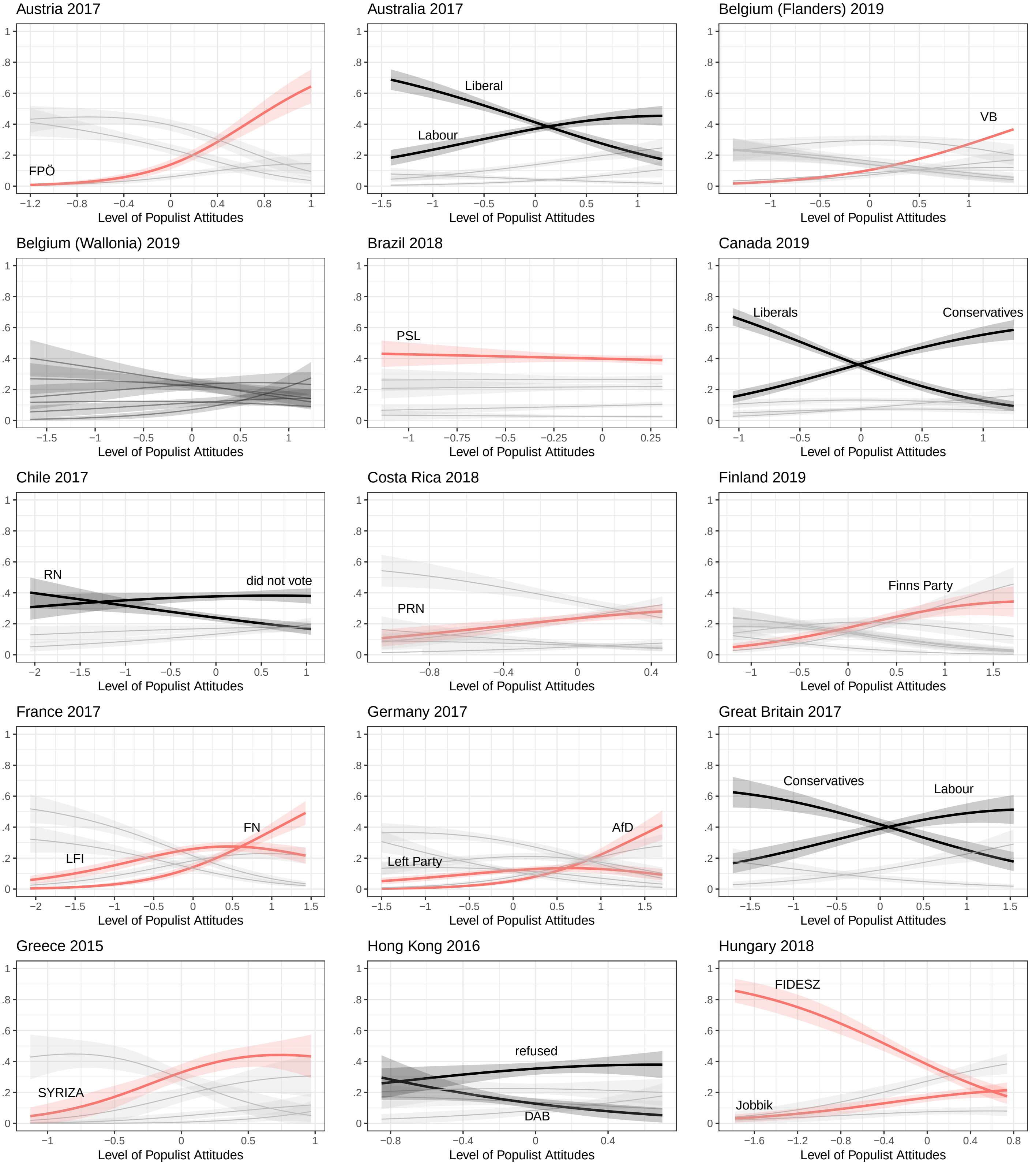

**Fig S2. Predictions of Populist Vote Across Countries (3 Dimensions) II**
Predictions of vote choice are based on multinomial structural equation models with 95% confidence intervals.

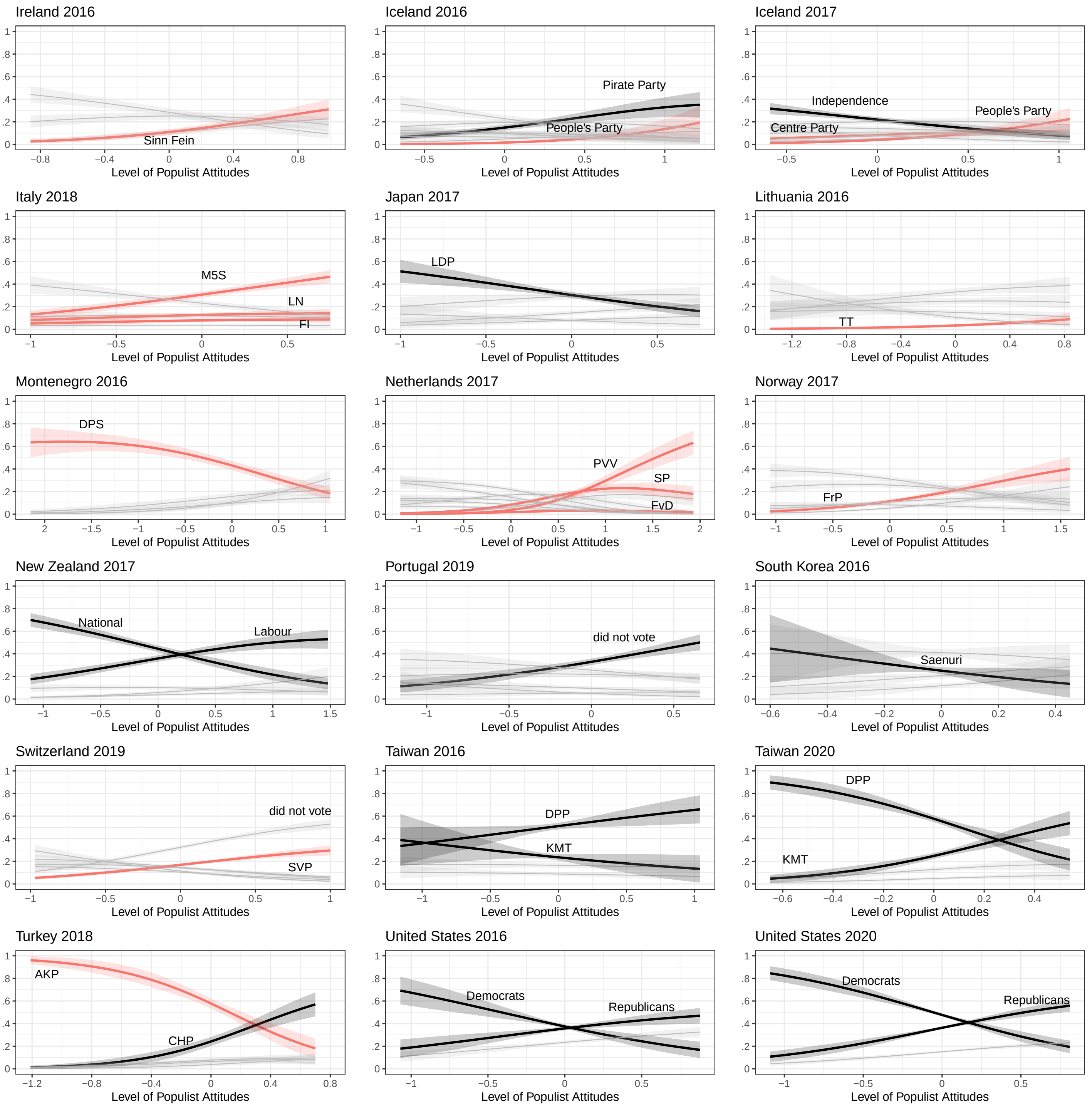

**Fig S3. Predictions of Populist Vote Across Countries (Goertz: 3 Dimensions) I** Predictions of vote choice are based on multinomial regression models with 95% confidence intervals.

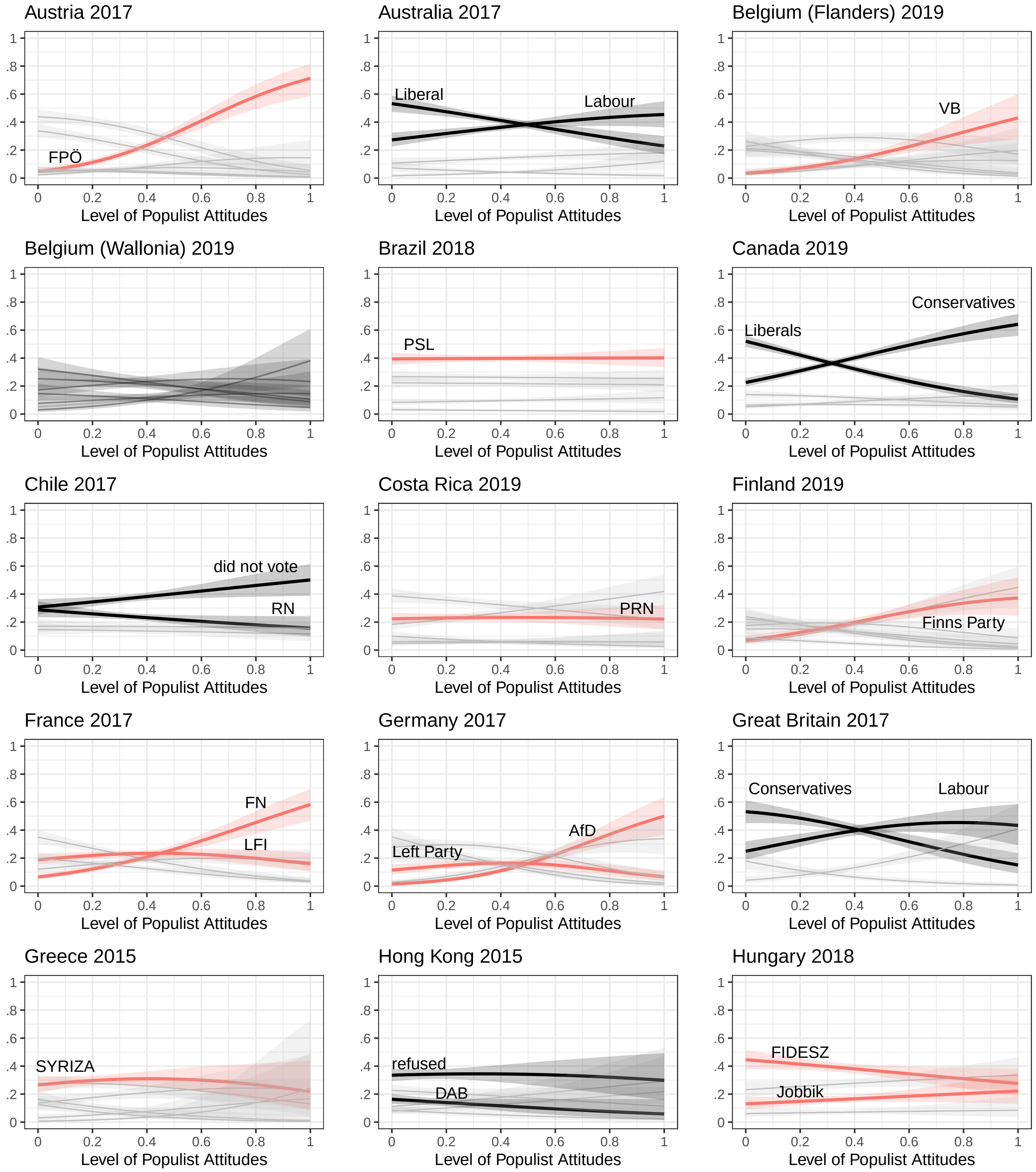

**Fig S4. Predictions of Populist Vote Across Countries (Goertz: 3 Dimensions) II** Predictions of vote choice are based on multinomial regression models with 95% confidence intervals.

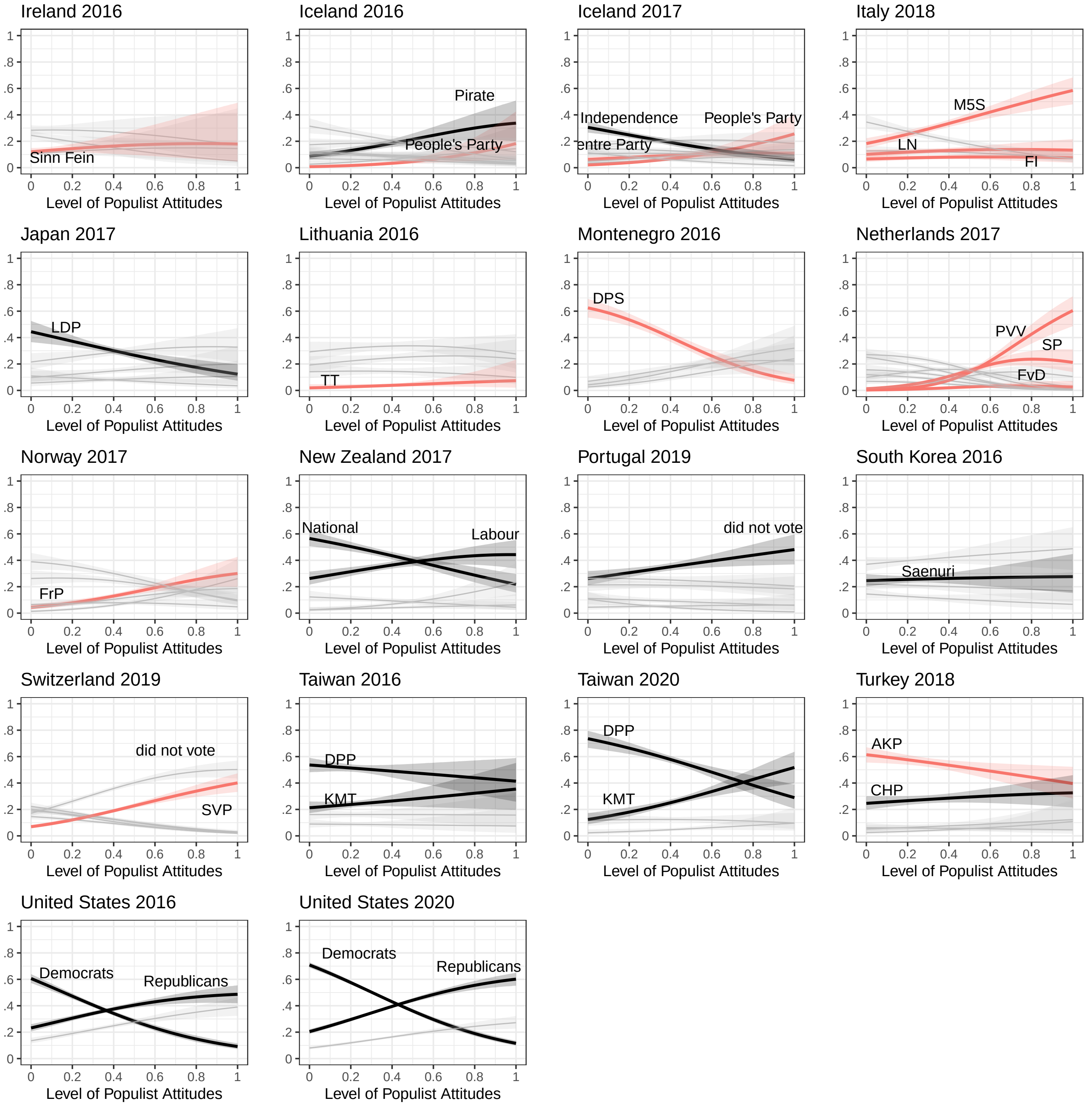

**Fig S5. Predictions of Populist Vote Across Countries (Goertz: Wuttke et al.) I** Predictions of vote choice are based on multinomial regression models with 95% confidence intervals.

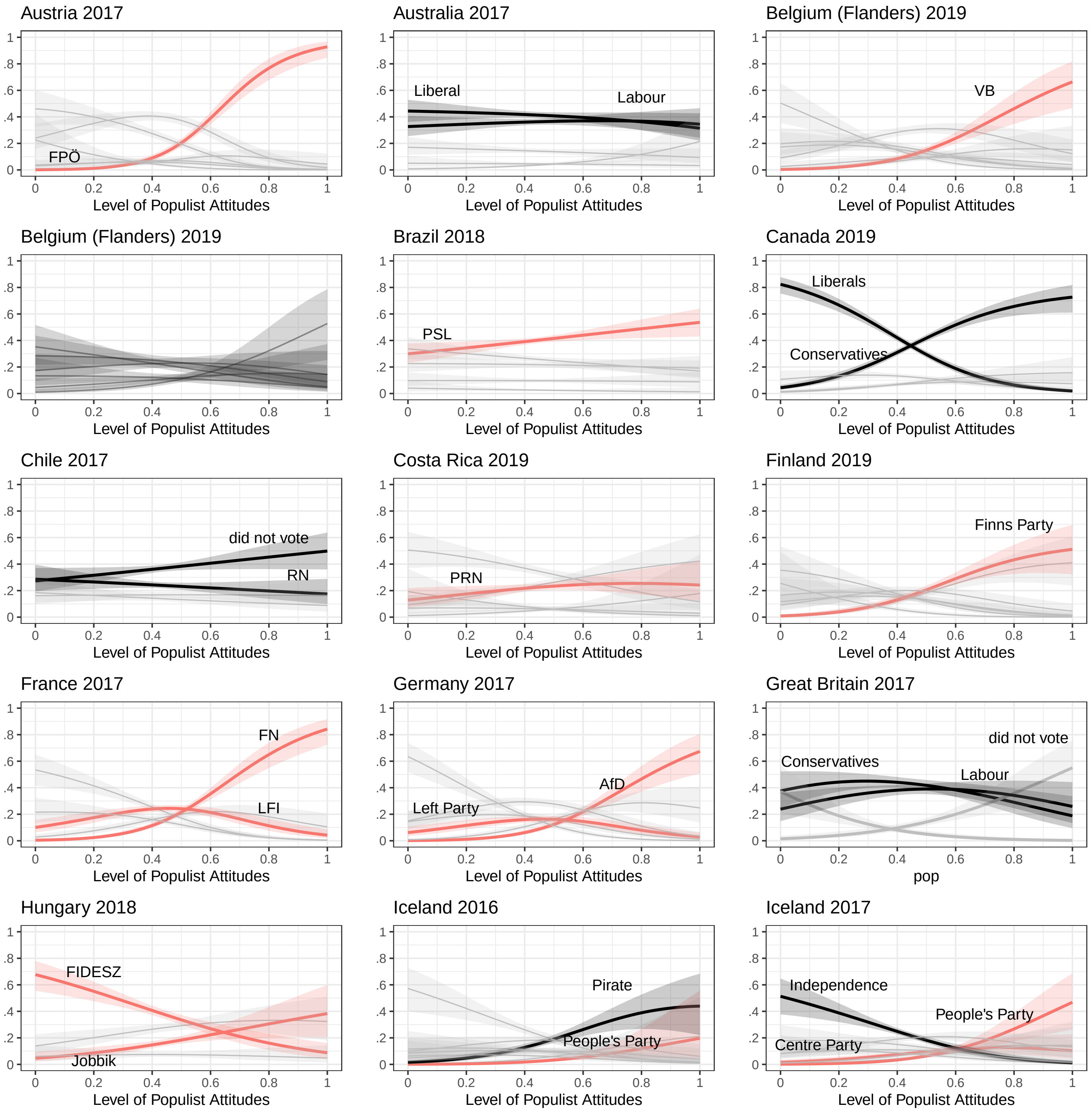

**Fig S6. Predictions of Populist Vote Across Countries (Goertz: Wuttke et al.) II** Predictions of vote choice are based on multinomial regression models with 95% confidence intervals.

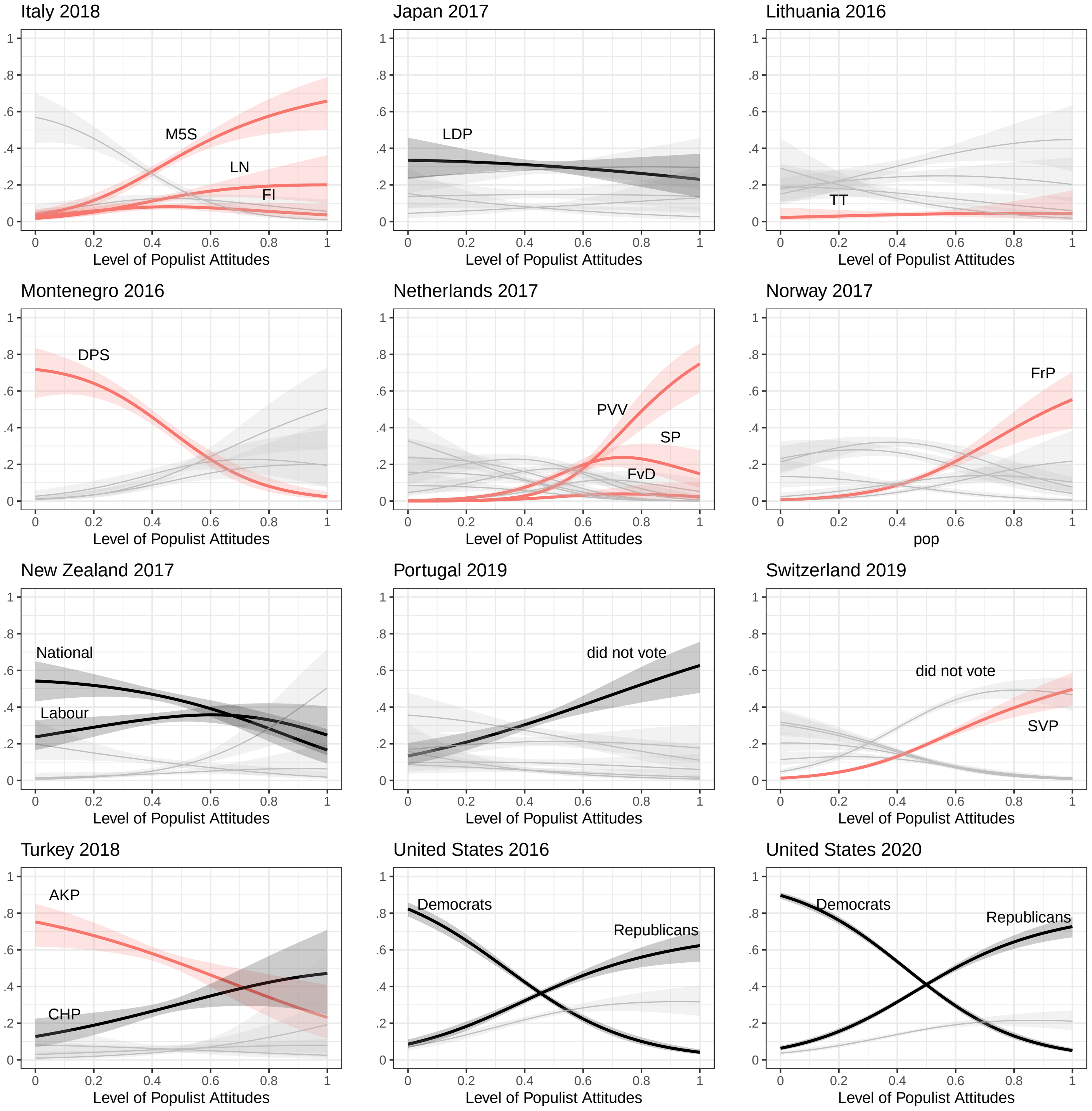

**Fig S7. Predictions of Populist Vote Across Countries (Goertz: Wuttke et al. without item E3007) I** Predictions of vote choice are based on multinomial regression models with 95% confidence intervals.

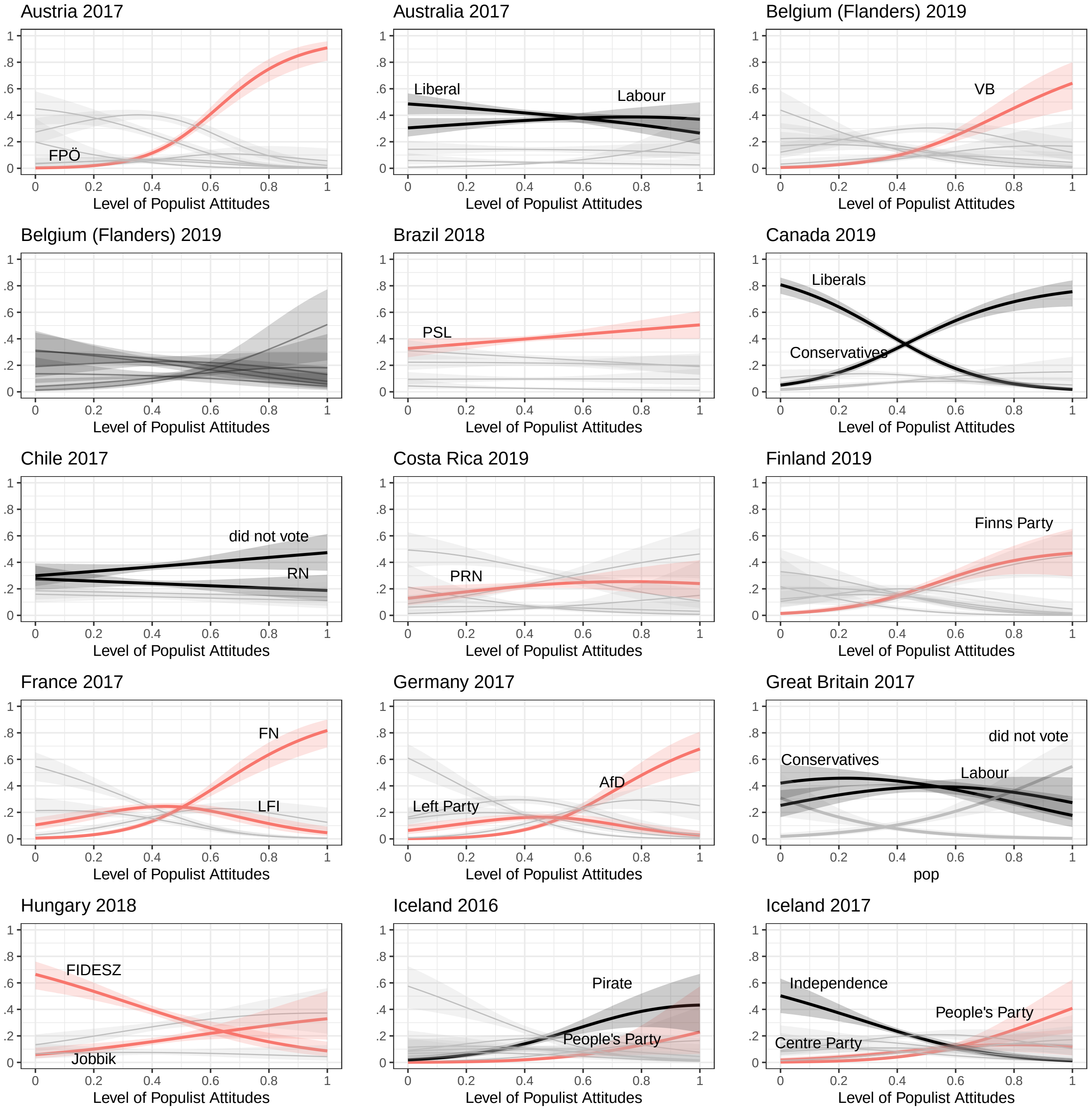

**Fig S8. Predictions of Populist Vote Across Countries (Goertz: Wuttke et al. without item E3007) II** Predictions of vote choice are based on multinomial regression models with 95% confidence intervals.

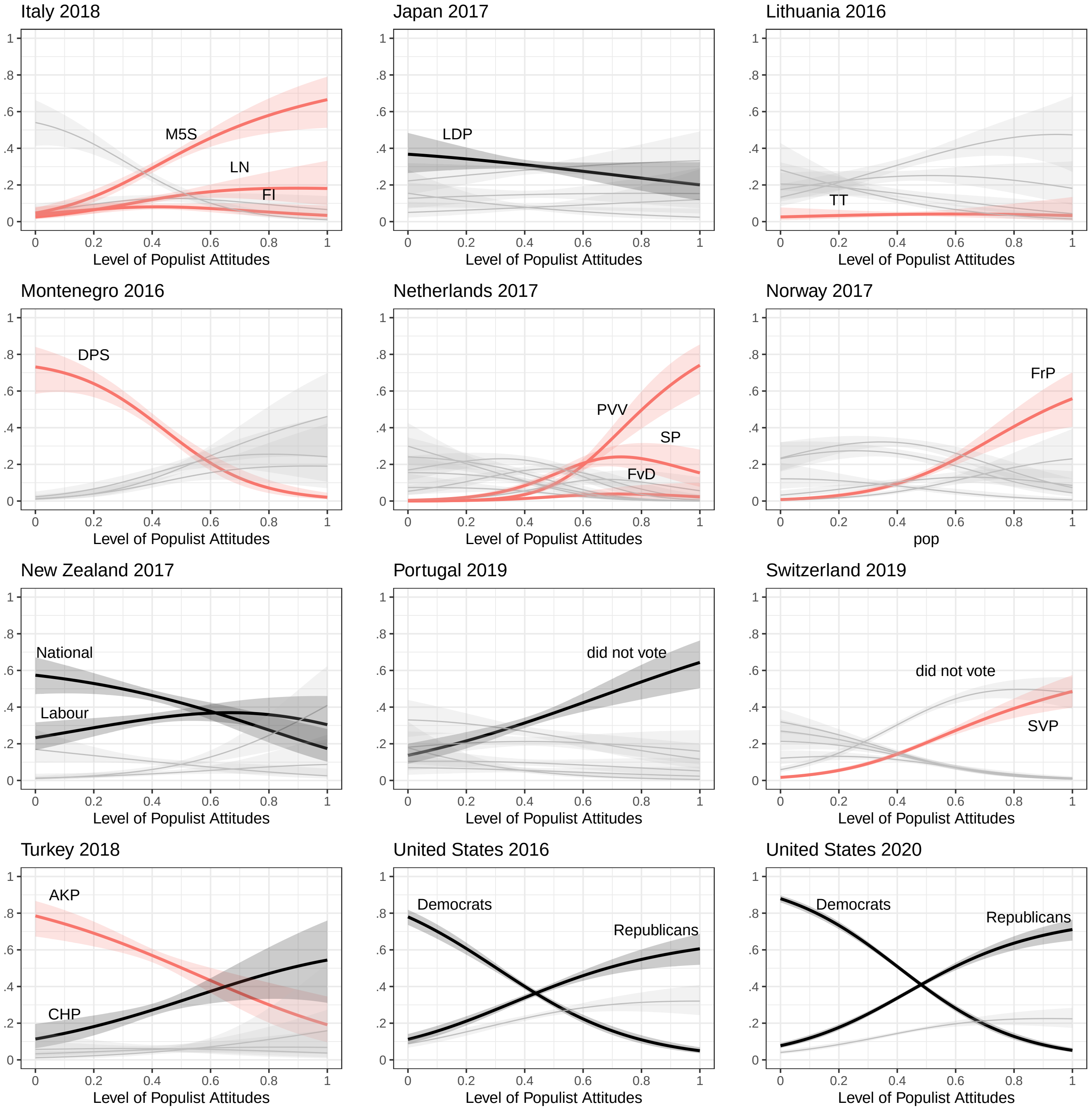

# Appendix B: Robustness Checks

## Greece (2016)

The first robustness check uses the Greek data from Castanho Silva et al.[1] who sampled 310 adults via CrowdFlower as part of a nine country survey between December 2016 and March 2017 (median age: 32 years, 55% females, median years of education: 15, median income decile: 3rd, mean left-right self-placement on a 1-9 scale, where 1 is the left: 5.2). At the time of the survey, SYRIZA has been the ruling party since the January 2015 elections. We estimate the effect of six different populist attitudes scales on vote choice for SYRIZA and find that only one of the scales (Stanley 2011) produces a slightly positive effect whereas all the other scales show no effect [1–6]. This provides further evidence that populist attitudes scales do not work as proposed whenever populist parties are in power.

**Table S4.** Question Wording Akkerman et al. Items

| Item | Wording |
|---|---|
| AKK1 | The politicians in the Greek Parliament need to follow the will of the people. |
| AKK2 | The people, and not politicians, should make our most important policy decisions. |
| AKK3 | The political differences between the elite and the people are larger than the differences among the people. |
| AKK4 | I would rather be represented by a citizen than by a specialized politician. |
| AKK5 | Elected offcials talk too much and take too little action. |
| AKK6 | What people call "compromise" in politics is really just selling out on ones principles. |

**Table S5.** Question Wording Castanho Silva et al. Items

| Item | Wording |
|---|---|
| CS-PPL1 | Politicians should always listen closely to the problems of the people. |
| CS-PPL2 | Politicians don't have to spend time among ordinary people to do a good job. |
| CS-PPL3 | The will of the people should be the highest principle in this country's politics. |
| CS-ANT1 | The government is pretty much run by a few big interests looking out for themselves. |
| CS-ANT2 | Government officials use their power to try to improve people's lives. |
| CS-ANT3 | Quite a few of the people running the government are crooked. |
| CS-MAN1 | You can tell if a person is good or bad if you know their politics. |
| CS-MAN2 | The people I disagree with politically are not evil. |
| CS-MAN3 | The people I disagree with politically are just misinformed. |

**Table S6.** Question Wording Elchardus and Spruyt Items

| Item | Wording |
|---|---|
| ES1 | The opinion of ordinary people is worth more than that of experts and politicians. |
| ES2 | Politicians should listen more closely to the problems the people have. |
| ES3 | Ministers should spend less time behind their desks, and more among the ordinary people. |
| ES4 | People who have studied for a long time and have many diplomas do not really know what makes the world go round. |

**Table S7.** Question Wording Schulz et al. Items

| Item | Wording |
|---|---|
| S-ANT1 | MPs in Parliament very quickly lose touch with ordinary people. |
| S-ANT2 | The differences between ordinary people and the ruling elite are much greater than the differences between ordinary people. |
| S-ANT3 | People like me have no influence on what the government does. |
| S-SOV1 | The people should have the final say on the most important political issues by voting on them directly in referendums. |
| S-SOV2 | The people should be asked whenever important decisions are taken. |
| S-SOV3 | The people, not the politicians, should make our most important policy decisions. |
| S-HOM1 | Ordinary people are of good and honest character. |
| S-HOM2 | Ordinary people all pull together. |
| S-HOM3 | Although the [NATIONALS] are very different from each other, when it comes down to it they all think the same. |

**Table S8.** Question Wording Stanley Items

| Item | Wording |
|---|---|
| STAN1 | The ordinary people are divided by many different values. |
| STAN2 | The people who belong to the political elite are divided by many different values. |
| STAN3 | Ordinary people are prevented from improving their lives by the actions of unaccountable elites. |
| STAN4 | Not all politicians are the same; some genuinely care about what the people want. |
| STAN5 | Democracy is about finding compromise between different interests and opinions. |
| STAN6 | Ordinary people are unable to make the correct decisions about the future of our country. |
| STAN7 | The majority of politicians are honest people. |
| STAN8 | Modern politics is in essence a struggle between the good, honest people and the evil elite. |

**Table S9.** Confirmatory Factor Analysis Models for Populist Attitudes Scales (Greece 2016)

| Country | N | RMSEA | SRMR | CFI | Avg. Loading | Min. Loading | Lowest Loading |
|---|---|---|---|---|---|---|---|
| Akkerman et al. | 254 | 0.077 | 0.038 | 0.948 | 0.570 | 0.507 | AKK3 |
| Castanho Silva et al. | 267 | 0.077 | 0.059 | 0.918 | 0.430 | 0.252 | CS-MAN2 |
| CSES | 254 | 0.056 | 0.037 | 0.969 | 0.537 | 0.369 | E3004_3 |
| Elchardus and Spruyt | 258 | 0.238 | 0.078 | 0.789 | 0.509 | 0.190 | ES4 |
| Schulz et al. | 246 | 0.045 | 0.037 | 0.974 | 0.629 | 0.484 | S-HOM3 |
| Stanley | 246 | 0.101 | 0.069 | 0.736 | 0.390 | 0.217 | STAN7 |

Shown are standardized loadings.

**Fig S9.** Predictions of SYRIZA Vote by Populist Attitudes Scales

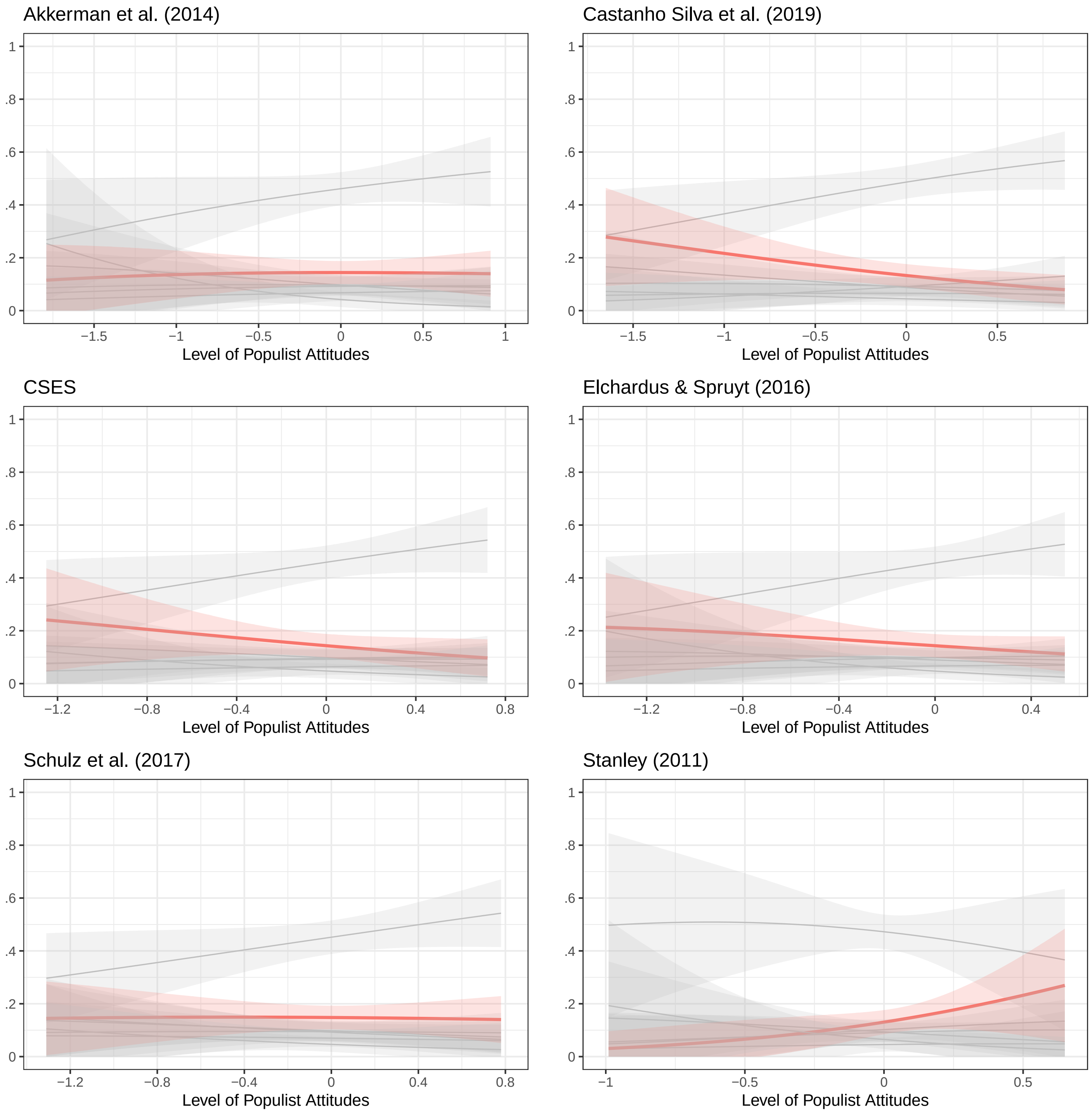

## Japan (2019)

As the Japanese and South Korean cases are both contested, we have not labelled these parties as populist. However, the LDP has been argued to have embraced some aspects of populist rhetoric and strategy, most notably under Junichiro Koizumi (Prime Minister from 2001-2006) but also under his successor Shinzo Abe (Prime Minister 2006-2007, 2012-2020) [7–9]), while both the Saenuri Party during the rule of Park Geun-hye and the leaders of the protest movement which ultimately deposed her from the presidency have been labelled populist by some scholars (see for example [10, 11]).

We fielded an online survey through a Yahoo Cloud panel in Japan in August 2019 in which we included the items of the Castanho Silva et al. and Schulz et al. scales along with questions on vote choice and political attitudes (N=1192). Prior research in Japan has indicated a disconnect between literature on populist leaders and parties and the actual voting behaviour of those voters classified as populists using existing survey scales [12]. Japan's ruling party, the Liberal Democratic Party (LDP), is an "edge case" in terms of the adoption of populist rhetoric and strategies; while the LDP cannot reasonably be bracketed alongside overtly populist parties such as Hungary's FIDESZ or Germany's AfD, the party (especially under recent influential leaders, Junichiro Koizumi and Shinzo Abe) is argued to have adopted a number of elements of populist rhetoric, embracing what Yoshida describes as a "Japanese style of populism" ([8]; see also [9, 13]. This strategy has been argued to have been effective in limiting the appeal of potential populist challengers from outside the party [14]. Japan therefore presents a complex test case for the existing scales which could show whether they work similarly when applied to an incumbent party which only partially meets the definitions of populism.

Our results were consistent with our analysis of the CSES Module 5 data in this case, with the same strong negative relationship between populist attitudes and vote for the LDP (see Fig S8). However, both scales had some issues regarding goodness of fit — the Castanho Silva et al. scale had poor fit metrics (CFI is less than .95 and RMSEA is over .06, per [15]) and its anti-elitism dimension correlated only weakly with the other two dimensions, while the Schulz et al. scale, though it scored well on fit measurements, had an unexpectedly negative loading on the populism factor for one of its dimensions (related to national homogeneity). These problems are consistent with our expectation that existing scales of populism are unsuited to complex cases, such as those where populist parties have entered government, or where existing mainstream parties have adopted populist strategies to appeal to certain voters. Our contention is not that the results for the Japanese case are "wrong", *per se*, since even most scholars who argue that the LDP has adopted populist rhetoric do not go so far as to claim that it's actually a populist party; rather, it's that when used in this context, the populist attitudes scales are not actually measuring what they purport to measure, and that their results may be meaningless or worse, actually misleading.

**Table S10.** Confirmatory Factor Analysis Models for Castanho Silva et al. and Schulz et al. scales (Japan 2019)

| Country | N | RMSEA | SRMR | CFI | Avg. Loading | Min. Loading | Lowest Loading |
|---|---|---|---|---|---|---|---|
| Castanho Silva et al. | 907 | 0.077 | 0.058 | 0.905 | 0.512 | 0.358 | PPL3 |
| Schulz et al. | 920 | 0.057 | 0.037 | 0.970 | 0.703 | 0.374 | ANT3 |

Shown are standardized loadings.

**Fig S10.** Predictions of LDP vote by Castanho Silva et al. and Schulz et al. scales

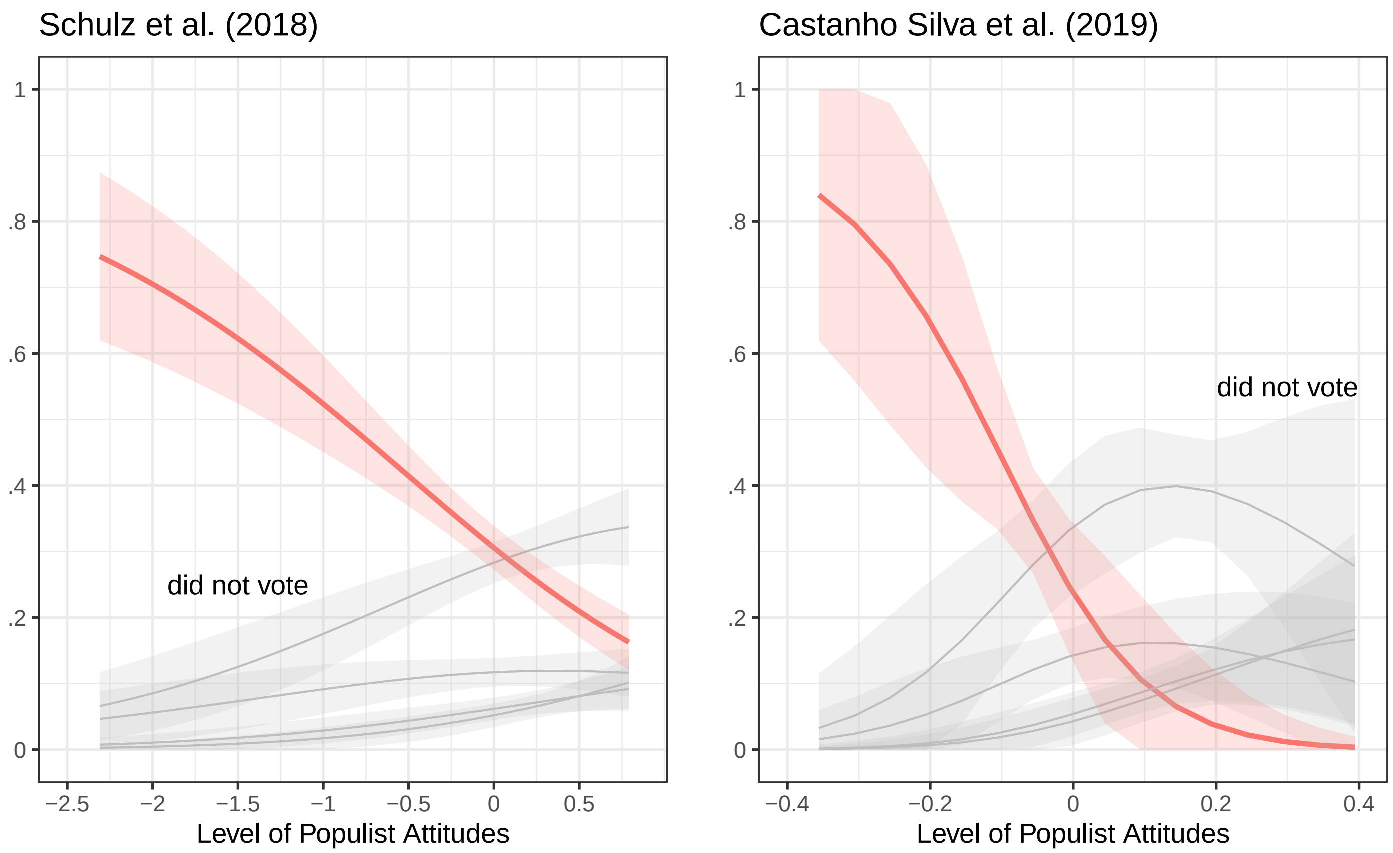

# References


1. Castanho Silva B, Andreadis I, Anduiza E, Blanuša N, Morlet Corti Y, Delfino G, et al. Public opinion surveys: a new scale. In: Hawkins KA, Carlin R, Littvay L, Rovira Kaltwasser C, editors. The Ideational Approach to Populism. Routledge; 2019. p. 150–171.
2. Akkerman A, Mudde C, Zaslove A. How Populist Are the People? Measuring Populist Attitudes in Voters. Comparative Political Studies. 2014;47(9):1324–1353. doi:10.1177/0010414013512600.
3. Comparative Study of Electoral Systems. CSES Module 5 Advanced Release 3: July 20, 2021 version; 2021.
4. Elchardus M, Spruyt B. Populism, Persistent Republicanism and Declinism: An Empirical Analysis of Populism as a Thin Ideology. Government and Opposition. 2016;51(1):111–133. doi:10.1017/gov.2014.27.
5. Schulz A, Müller P, Schemer C, Wirz DS, Wettstein M, Wirth W. Measuring Populist Attitudes on Three Dimensions. International Journal of Public Opinion Research. 2018;30(2):316–326.
6. Stanley B. Populism, nationalism, or national populism? An analysis of Slovak voting behaviour at the 2010 parliamentary election. Communist and Post-Communist Studies. 2011;44(4):257–270. doi:10.1016/j.postcomstud.2011.10.005.
7. Kobori M. Populism as Rhetorical Politics in Britain and Japan:"Devil take the hindmost". Ritsumeikan Law Review. 2013;30:107–22.
8. Yoshida T. Populism "made in Japan": A new species? Asian Journal of Comparative Politics. 2019;5(3):288–299. doi:10.1177/2057891119844608.
9. Fahey RA, Hino A, Pekkanen RJ. Populism in Japan. In: Pekkanen RJ, Pekkanen SM, editors. The Oxford Handbook of Japanese Politics. Oxford University Press; 2021. doi:10.1093/oxfordhb/9780190050993.013.18.
10. Kim H. "Spoon Theory" and the Fall of a Populist Princess in Seoul. The Journal of Asian Studies. 2017;76(4):839–849. doi:10.1017/S0021911817000778.
11. Shin GW, Moon RJ. South Korea After Impeachment. Journal of Democracy. 2017;28(4):117–131. doi:10.1353/jod.2017.0072.
12. Hieda T, Zenkyo M, Nishikawa M. Do populists support populism? An examination through an online survey following the 2017 Tokyo Metropolitan Assembly election. Party Politics. 2019;doi:10.1177/1354068819848112.
13. Lindgren PY. Developing Japanese Populism Research through Readings Of European Populist Radical Right Studies: Populism As An Ideological Concept, Classifications Of Politicians And Explanations For Political Success. Japanese Journal of Political Science. 2015;16(4):574–592. doi:10.1017/S1468109915000328.
14. Warren D. Understanding Japanese Democracy in a Populist Age. Asian Affairs. 2019;50(3):283–295. doi:10.1080/03068374.2019.1644029.
15. Hu L, Bentler PM. Cutoff Criteria for Fit Indexes in Covariance Structure Analysis: Conventional Criteria Versus New Alternatives. Structural Equation Modeling: A Multidisciplinary Journal. 1999;6(1):1–55. doi:10.1080/10705519909540118.
