# Supplementary material for: How populist attitudes scales fail to capture support for populists in power
Source: PLoS One. 2021 Dec 31;16(12):e0261658. doi: 10.1371/journal.pone.0261658 (PMC8719679; doi:10.1371/journal.pone.0261658)
Supplement: Attachment — Submitted filename: response_letter.pdf [file pone.0261658.s002.pdf]

RESPONSE LETTER

Dear editors and reviewers,

Thank you very much for giving us the opportunity to revise and resubmit our manuscript "How populist attitudes scales fail to capture support for populists in power".

First of all, we would like to thank you and the reviewers for the positive feedback as well as the detailed comments and suggestions. The reviewers were clearly very knowledgeable of the topic and have offered a number of thoughtful points to further improve the manuscript. We have taken their comments very seriously and tried our best to accommodate all suggestions. In this memo we outline in detail how we have addressed each individual comment by the reviewers.

We hope that this thoroughly revised manuscript now meets your and your readers' demands.

REVIEWER 1

COMMENT 1.1

Even though I think that the main observation (how voters react to populists in power) raises many questions that are worth thinking about, I see the contribution of this manuscript not so much as trying to enlighten these questions in a substantive matter. Rather, the contribution is practical and methodological, advising scholars of populist attitudes that their scales work differently depending on whether populists are in power. In doing so, this paper is likely more than the beginning (even though

similar work already exists, eg. Castanho Silva 2019) than the end of this line of research and the study helps other populism researchers avoid consequential mistakes.

Still, my most significant reservation concerns this substantive dimension of the observed phenomena: The authors interpret the observed and indeed very interesting phenomena of varying associations between populist parties and populist attitudes across countries largely as evidence of measurement error: Populist citizens are still considered populist but under certain conditions the scale no longer achieves to capture these sentiments. Yet, upon closer inspection there is not much evidence given for this interpretation in contrast to other conceivable interpretations: It is also possible that previously populist citizens have simply changed their minds after their preferred populist parties took power and they are no longer populist. Many authors consider populism as a redemptive force and maybe populists have now gotten what they wanted, so there is no longer the need for populist attitudes (populism as a thermostatic attitude). Answering this question requires engaging with conceptual questions on the exact definition of what anti-elitism means and which types of actors may be considered here (eg political or non-political actors — to me it is not self-evident that populism must necessarily also include non-political actors as it is a politics-focused concept). While there is no need to engage with these questions in the empirical section of the article, I would urge the authors to at least contemplate these questions and discuss them either as limitations of their study or as potential avenues for further research. Primarily, I would ask the authors to consider on what evidential or conceptual bases they think the conclusion is warranted that we are dealing with a measurement problem as opposed to actual changes in attitudes.

RESPONSE 1.1

We agree that the paper was too quick to dismiss the possibility of populist attitudes being thermostatic and thus changing rapidly once populist leaders are elected, which is certainly an alternative explanation for our findings. We still believe that what we have observed is a measurement problem, but have tried to justify this belief more effectively by engaging with these arguments in both the introduction (pages 2, 4, and 6–7) and the discussion section (pages 19–20). While other reasonable interpretations are possible, we argue that the observation that populist leaders in power often simply shift their anti-elite rhetoric to different elite targets rather than moving away from populist rhetorical strategies more broadly suggests that populist attitudes (at least as commonly defined, though perhaps not with a stricter political populism definition) still persist among their voters. The continuing strategic value of populist rhetoric to elected leaders may indicate that populist attitudes are more persistent than thermostatic, or perhaps that although thermostatic, their underlying demands have not been satisfied merely by the election of a populist party. Either way, we believe that many researchers would expect populist attitudes scales to continue to capture such attitudes among supporters of elected populists, making it important to elucidate the issues with this kind of measurement.

COMMENT 1.2

The authors conduct CFA to test whether the data fit populist attitudes as described by Wuttke et al. But Wuttke et al. conceive of populist attitudes as non-compensatory which does not imply a correlation between the subcomponents of populism (even though a strong correlation between the indicators of each subcomponent

is expected). But the presented CFA likely specified such a correlation between the subdimensions and the strength of that correlations determine the reported goodness of fit indices — if I am not mistaken. Hence, I am wondering whether the model and the conclusion fit the concept specification in this case.

RESPONSE 1.2

That is correct, although this has not been our intention. We reported the CFA for mere comparison with the Casthano Silva et al items of the CSES, as there has not been such a comparison of this operationalization of the items so far. However, Reviewer 1 is right that this might be perceived wrongly by the reader so that we present an operationalization of the Wuttke et al items which we later on actually don't use. This might be confusing and we reworked this section. We now moved the table into the appendix (now Table S3) and discuss it only briefly for comparison with the Castanho Silva et al. version, as we think that it still has merit for some readers. That said, we specifically state that this is not the way it was intended for use by Wuttke et al., nor is it used this way by us later on.

COMMENT 1.3

I was first wondering whether it is a reasonable choice to conduct a multinomial (instead of a binary) regression, but it is an excellent choice as proven with the very helpful and informative Figure 1!

RESPONSE 1.3

Thank you very much!

COMMENT 1.4

The manuscript states that "we coded populist parties along the schema provided by the PopuList [50] except for borderline cases." Which were these borderline cases, why have you decided not to follow PopuList in these cases and how does this change the substantive conclusions drawn from the empirical analysis?

RESPONSE 1.4

Initially, we did not highlight borderline cases like the Centre Party in Iceland or the Forza Italia in Italy in red because we focused on the ones which were more prominent, like the M5S or Lega Nord in Italy. To rule out potential arbitrariness, we now highlight all parties in red which are considered to be populist by the PopuList. However, we excluded all parties that received less than 5% during the respective national election for computational reasons (too low sample size for estimation). The overview in Table S1 has been adjusted accordingly.

COMMENT 1.5

On page 16, it reads that the second column of Table 1 explains the three dimensions of populist attitudes, but that column gives only the question wordings.

RESPONSE 1.5

You are correct, it is the third column. We changed it accordingly.

COMMENT 1.6

Please include the figures in the next version of the manuscript right in the text

as the manuscript is otherwise hard to navigate. More importantly, the figures are extremely pixelated right now

RESPONSE 1.6

We agree that including figures in the text may have been easier to read and review. Yet, it was the journal's requirement to remove them from the text and upload them separately (in .tiff, not .pdf). The submission site then adds pixelated figures at the end of the manuscript. Please access the high quality figures by clicking the link in the upper right-hand corner and download them. This is also specified here: https://everyone.plos.org/2011/02/11/ask-everyone-figure-files-in-your-merged-pdf/

COMMENT 1.7

The replication data are (not yet?) uploaded on the linked dataverse

RESPONSE 1.7

We are very sorry that the files had not been uploaded yet. We were not sure how to handle this during the PLOS ONE workflow, as we did not want to violate the blinded review process. The files are now uploaded to the Harvard Dataverse and can be reviewed. Again, please accept our apologies for this delay in making the materials available.

REVIEWER 2

COMMENT 2.1

Populist Attitudes and Populist Voting

The paper convincingly shows how the populist attitude scales behave differently in different political contexts. To this end, the authors show that the predictive quality of populist attitudes for populist voting differs when populist party is in office. While I agree that this is a good way to get a grasp of the differential substantive meaning of populist attitude scales, I do not agree that the central aim of the concept of populist attitudes is that it predicts populist voting, as the authors suggest on p. 6. Rather, populist attitude scales have a much broader application. Populism (and populist attitudes) can be considered to be a view on political representation (Urbinati 2019). This is not to say that populist attitudes haven't been used or validated by measuring their effect on populist voting. It is simply that the concept's utility is broader. I think the paper would be stronger if it reflects on this issue a bit.

RESPONSE 2.1

It was not our intention to claim that populist attitudes scales are primarily designed to predict voting behaviour — populism is a wide-reaching concept which we would argue has important implications not only for an individual's views of political representation but of society more broadly. We have expanded on our discussion of the nature of populist attitudes, particularly in the Introduction section, and have

edited the section you refer to (pages 7 and 8) to make it more clear that we view voting behaviour as a potentially useful way to validate populist attitudes, rather than viewing populist attitudes as simply being a predictor of voting.

COMMENT 2.2

Variations of Populist Attitudes Scales.

Although it is beyond the author's discretion, it is a shame that the CSES failed to include the original six items as proposed by Akkerman et al. Instead the authors have to rely on the other items included in the CSES. As the authors also acknowledge, the item E3004_1 on compromise does not fit the Manichean category. A Manichean world view implicates that politics is a struggle between good and evil. While both the rejection of compromise and the Manichean worldview have a anti-pluralist component, the Manichean worldview sees the struggle between opposing camps as an inherently moral one. As such, I would suggest that E3004_1 should be labelled as measuring Manichean worldview, even if Castanho Silva et al (2020) proposed this. I agree with Wuttke et al (2020) that it rather fits a representation-related dimension. Interestingly, also the original AKkerman et al. scale failed to find a good item to capture the Manichean worldview.

RESPONSE 2.2

We agree that the item does not adequately reflect the Manichean dimension of populism for the reasons mentioned. We now describe it as "challenge to representative democracy (which shares the anti-pluralist component of a Manichean worldview)". Furthermore, we elaborate further in a footnote that item E3004_1

does not properly reflect the moral struggle between two opposing groups.

COMMENT 2.3

In the Wuttke et al. (2020) CSES scale, an item about corruption by politicians is included. I believe that corruption perceptions cannot be equated or subsumed under 'populist attitudes'. As Meijers and Zaslove argue in their critique of the Chapel Hill Expert Survey measuring populism (also) as party positioning on corruption, populists do not aim to fight actual corruption (i.e. misuse of power for private gain). Rather, as Taggart (2018) argues, participating in politics is corruptING. As such, I would remove the item from the main analysis. This might affect the results as Goertzian approach proposes to use the minimum score for all dimensions/components. The full CSES scale proposed by Wuttke et al can be shown in the appendix.

RESPONSE 2.3

Thank you very much for pointing this out. It is really a pity that the CSES committee decided against the implementation of a previously tested scale for populist attitudes. Thus, there are many potential ways on how to operationalize them. Our idea was to use the two already existing ways of operationalizing populist attitudes in the CSES (according to Castanho Silva et al. and Wuttke et al.) which gives us some comparability to other studies. That said, we agree that including the corruption item is not an adequate way of measuring populist attitudes. We now elaborate on this in the text on page 9. We also ran all models again using an operationalization without the corruption item. The results are basically identical to those with the item (r=0.96 between the two versions of the scale). However, we decided to keep

the original Wuttke et al. version in the main text for comparability and added the new version to the appendix (Figures S7 and S8). We hope that these changes are acceptable.

COMMENT 2.4

A strength of the paper is that it replicates the analysis for Greece and Japan by testing different types of scales including the original Akkerman et al (2014) scale and the Schulz et al (2017) scale. Yet, I think the paper would benefit from a more in-depth discussion on how these scales differ (and, importantly, are similar) in the "Data & Operationalization" section. Currently, this is only briefly discussed at the end.

RESPONSE 2.4

The points of comparison and contrast between different populist attitudes scales are discussed in a great deal of depth in Castanho Silva et al. 2019 (whose data for Greece we use with permission in this paper), with an even broader set of scales being considered in Castanho Silva et al. 2020. We definitely agree, however, that this paper would benefit from some more detail about the differences between the scales we tested, and have added an overview of the conceptual differences between the scales on Page 11. We note that despite very different approaches to operationalizing populism, these scales all yield similar results in our tests, which we attribute to their shared assumption that the term "elites" refers exclusively to political elites.

COMMENT 2.5

Discussion: What Now?

While I understand that it is beyond the scope of this paper to develop and test a new scale of populist attitudes which circumvents the problems diagnosed with current populism scales, I think the authors could do more to discuss what such a new populist scale in future research should look like. The authors' main argument is that currently the populist attitudes scales prime respondents to think about political elites only. Yet, how could this issue be addressed? Removing all references to politicians and only refer to elites would render the questions perhaps too vague and too complex. Would everyone understand what (and who) elites are? It would be great if the paper could propose ways in which one could measure populist attitudes in cross-national fashion independent of the political context.

RESPONSE 2.5

We appreciated this comment as this challenge has occupied a great deal of our attention, and testing hypotheses in this area forms a major part of our future research agenda. While there are no easy answers to this question, we have added some discussions of the problem to the conclusions section (pages 20–21) in order to outline the strengths of weaknesses of different possible approaches. Wording the questions to refer vaguely to elites risks under-specification and runs into enormous problems in translation, as even the best translation of this term can have very different nuances in other languages. However, asking about specific possible targets of anti-elite sentiment risks over-specification, and some elite groups may be very specific to a given country or region. Much further work is required to find a way

through this issue that works effectively cross-nationally, but we hope that the outline of the problem space we have added to the paper will be of use to readers.

References

Akkerman A, Mudde C, Zaslove A. How Populist Are the People? Measuring Populist Attitudes in Voters. Comparative Political Studies. 2014;47(9):1324–1353.

Castanho Silva B, Andreadis I, Anduiza E, Blanuša N, Morlet Corti Y, Delfino G, et al. Public opinion surveys: a new scale. In: Hawkins KA, Carlin R, Littvay L, Rovira Kaltwasser C, editors. The Ideational Approach to Populism. Routledge; 2019. p. 150–171.

Castanho Silva B, Jungkunz S, Helbling M, Littvay L. An Empirical Comparison of Seven Populist Attitudes Scales. Political Research Quarterly. 2020;73(2):409–424.

Taggart P. Populism and 'unpolitics'. In: Fitzi G, Mackert J, Turner BS, editors. Populism and the Crisis of Democracy. Volume 1: Concepts and Theory. London and New York: Routledge; 2018. p. 79–87.

Meijers MJ, Zaslove A. Measuring Populism in Political Parties: Appraisal of a New Approach. Comparative Political Studies. 2021;54(2):372–407.

Schulz A, Müller P, Schemer C, Wirz DS, Wettstein M, Wirth W. Measuring Populist Attitudes on Three Dimensions. International Journal of Public Opinion Research. 2018;30(2):316–326.

Urbinati N. Me the People: How Populism Transforms Democracy. Harvard University Press; 2019.

Wuttke A, Schimpf C, Schoen H. When the Whole is Greater Than the Sum of its Parts: On the Conceptualization and Measurement of Populist Attitudes and Other Multi-dimensional Constructs. American Political Science Review. 2020;114(2):356–274.
